# A Comparison of Cut Points for Measuring Risk Factors for Adolescent Substance Use and Antisocial Behaviors in the U.S. and Colombia

**DOI:** 10.3390/ijerph18020470

**Published:** 2021-01-06

**Authors:** Eric C. Brown, Pablo Montero-Zamora, Francisco Cardozo-Macías, María Fernanda Reyes-Rodríguez, John S. Briney, Juliana Mejía-Trujillo, Augusto Pérez-Gómez

**Affiliations:** 1 Department of Public Health Sciences, Miller School of Medicine, University of Miami, Miami, FL 33136, USA; pxm527@miami.edu (P.M.-Z.); foc9@miami.edu (F.C.-M.); 2 School of Psychology, Universidad El Bosque, Bogotá 110121, Colombia; mafereyes439@gmail.com; 3 Social Development Research Group, School of Social Work, University of Washington, Seattle, WA 98115, USA; jsb@uw.edu; 4 Corporación Nuevos Rumbos, Bogotá 110111, Colombia; jmejia@nuevosrumbos.org (J.M.-T.); aperez@nuevosrumbos.org (A.P.-G.)

**Keywords:** risk factors, adolescent substance use, youth antisocial behaviors, Communities That Care, Comunidades Que se Cuidan

## Abstract

As the identification and targeting of salient risk factors for adolescent substance use become more widely used globally, an essential question arises as to whether U.S.-based cut points in the distributions of these risk factors that identify “high” risk can be used validly in other countries as well. This study examined proportions of youth at “high” risk using different empirically derived cut points in the distributions of 18 measured risk factors. Data were obtained from large-scale samples of adolescents in Colombia and the United States. Results indicated that significant (*p* < 0.05) differences in the proportions of “high” risk youth were found in 38.9% of risk factors for 6th graders, 61.1% for 8th graders, and 66.6% for 10th graders. Colombian-based cut points for determining the proportion of Colombian youth at “high” risk were preferable to U.S.-based cut points in almost all comparisons that exhibited a significant difference. Our findings suggest that observed differences were related to the type of risk factor (e.g., drug specific vs. non-drug specific). Findings from this study demonstrate the need for collecting large-scale national data on risk factors for adolescent substance use and developing country-specific cut points based on the distributions of these measures to avoid misidentification of youth at “high” risk.

## 1. Introduction

Substance use and addiction is still one of the most important public health problems facing youth worldwide [1]. From a prevention science perspective [2], the identification and targeting of risk factors, as precursors and causal mechanisms for adolescent substance use, has been shown to be the predominant and most effective way to address this problem [3,4,5]. Despite a wealth of information [6] and multiple registries of efficacious and effective programs to prevent youth substance use [7], uniform procedures to identify and address salient risk factors for youth substance use in communities are sorely lacking. Prevention program implementation and technical assistance systems that guide community coalitions and local agencies toward effective action [8,9] often rely on the community diagnosis model [10] to connect prevention initiatives with appropriate evidence-based programs.

**Figure 1:**
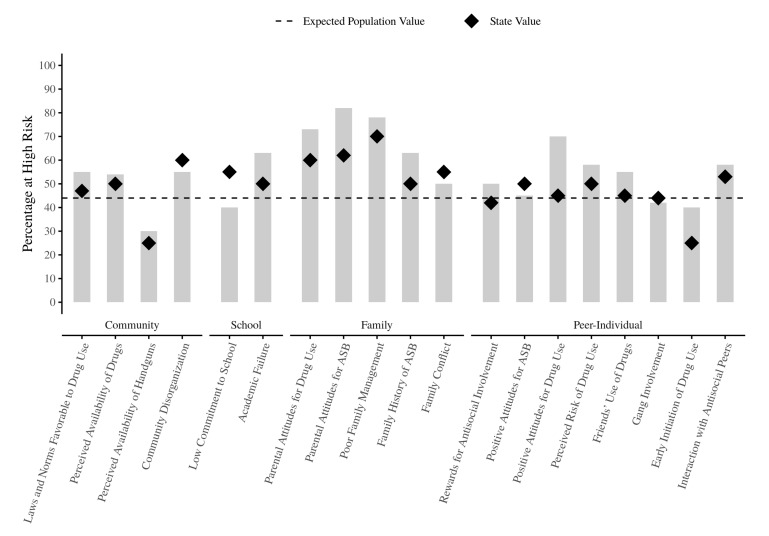
Risk Factor Profile for a School.

The community diagnosis model suggests that risk factor data, collected via youth surveys administered in schools or communities, can be used to create risk factor profiles that identify the proportions of youth in the school or community that are at “high” risk for substance use and other youth antisocial behaviors [11]. Such risk factor profiles have been shown to be valid and useful tools for prevention programming by community coalitions [12]. A hypothetical example of a risk factor profile for a specific school is shown in Figure 1 where each bar in the figure shows the percentage of youth identified as being at “high” risk for each measured risk factor, based on a particular decision rule for determining what “high” risk is. In this example, the most elevated risk factors are Poor Family Management (in the family domain) and Parental Attitudes Favorable for Drug Use (in the family and peer-individual domains). Ideally, elevated risk factors should be linked subsequently to appropriate evidence-based prevention programs following an action plan derived from a local community prevention coalition or local agency. Often, school district, county, and state averages are included in the profiles for comparative purposes. Recent extensions of the community diagnosis model are being applied in the international arena for national [13] and cross-national [14,15,16] comparisons.

Strengths of the community diagnosis model include having a source of data that is derived from the perceptions of youth in the community (which might be different than the perceptions of risk by adults in the community), and the ability for “high” risk to be easily calculated and interpreted from psychometrically validated risk factor measures. However, the community diagnosis model is not without its limitations. Feinberg et al. [10] note that time lags between data collection and action plan formation (which can take years), uniform weighting of risk factors in prevention planning (i.e., some risk factors correlate more strongly with outcomes than others), and comparisons of elevated risk factors are usually made vis-a-vis other risk factors in a profile and not against a “gold standard” level of risk.

An important caveat to this model is the need for valid estimation of the proportion of youth at “high” risk for each risk factor that makes up the risk profile. As the use of prevention systems that rely on the community diagnosis model are increasing worldwide, an essential question arises as to the international generalizability of cut points in the distributions of risk factor measures to identify “high” risk populations. To the extent that prevention programs and strategies are developed to confront specific risk factors for adolescent substance use, the efficacy of these interventions may be undermined by incorrect identification of relevant risk factors in a community, school, or other setting. This study examined the cross-national validity of U.S.-based cut points in measures of risk factors for adolescent substance use by comparing proportions of youth at “high” risk using different empirically derived cut points obtained from large-scale samples of youth in Colombia and the United States.

## 2. Methods

### 2.1. Study Design

This study is a secondary analysis of two large-scale epidemiologic data sets of youth risk factors for youth substance use, collected from youth in the United States and Colombia, respectively. U.S. data were provided by Bach Harrison L.L.C. (http://www.bach-harrison.com/), a survey research and evaluation firm that specializes in the analysis of data related to youth-based risk and protective factors. Colombian data were provided by the Corporación Nuevos Rumbos (https://www.nuevosrumbos.org/), a research and evaluation organization that specializes in the prevention of substance abuse in youth and adult populations, and the Universidad El Bosque, in Bogotá, Colombia (https://www.unbosque.edu.co/). All data sources relied on a common survey instrument, the Communities That Care Youth Survey (CTC-YS) [17], which was developed at the University of Washington and adapted in Colombia [18] to provide a validated community-based assessment tool for identifying salient risk and protective factors for youth health and behavior problems (e.g., substance use, violent and delinquent behaviors, risky sexual behaviors, and mental health problems) as part of the Communities That Care (CTC) prevention system.

CTC [19] is an evidence-based prevention “operating system”, designed to mobilize a community for effective prevention of youth antisocial behaviors. Experimental and quasi-experimental trials of CTC have demonstrated the efficacy of the system to impact targeted risk and protective factors [4,20] and related youth health and behavior problems (i.e., alcohol and drug use, violent and delinquent behaviors) [21,22]. In the U.S., hundreds of communities in dozens of states have implemented CTC, and many countries abroad (i.e., Australia, Brazil, Canada, Chile, Colombia, Croatia, Germany, India, Mexico, the Netherlands, and Sweden) have adapted the CTC system or the CTC-YS for use [20,23]. For example, in 2012, the Corporación Nuevos Rumbos began to implement an adapted version of CTC (called Comunidades Que se Cuidan) as a potential framework for a national system of youth substance use prevention in Colombia [24].

An important element of the CTC system is the targeting of salient risk and protective factors for intervention via high-fidelity implementation of a set of community-tailored prevention programs and strategies. The original empirically-identified formula for optimal bifurcation in the grade-specific distributions of risk and protective factor measures used in the CTC-YS was developed by Arthur et al. [11] with cut point calculated at *Md* + (0.15 × MAD), where MAD = mean absolute deviation about the median (*Md*), a measure of central tendency of a distribution around its median [25]. Under the assumption of a Gaussian distribution for a risk factor, this equation would yield an estimated 44% of youth above the “high” risk cut point. Minor adjustments to this formula have been made to account for the coarseness in the distributions of risk factor measures with a small number of items; however, the original Arthur et al. [11] equation stands as the basis for the calculation of proportions of youth at high risk using the CTC-YS and similar measures.

### 2.2. Measures

The CTC-YS [17] is a youth self-administered, paper-and-pencil questionnaire designed to be completed in a 50-min classroom period. The survey includes questions on student demographic characteristics (i.e., age, gender, race and ethnicity, family composition, and parental education); lifetime and 30-day measures of alcohol, marijuana, cigarette, and other drug use; heavy episodic drinking (i.e., five or more drinks in a row); past-year delinquency; and risk and protective factors in community. The U.S.-based CTC-YS has identified 28 potential risk factors for potential inclusion in its school-based surveys [26], however, different versions of the CTC-YS used in the U.S. and abroad contain different numbers and sets of risk and protective factors. From the data provided by Bach Harrison L.L.C. and the Corporación Nuevos Rumbos, we identified 18 risk factors across community, school, family, and peer-individual domains that had comparable items and response options. A list of risk factors, items, and country-specific cut points, are included in Annex I (Found in supplementary files, Table S1). A comparison of Spanish-language cultural equivalency between the English U.S.-based survey and the Spanish Colombian-based survey was conducted by the study investigative team who were fluent in both languages. Examinations of CTC-YS scale reliability for both the U.S.-based [17,26] and Colombian-based [18] risk and protective factor measures have demonstrated acceptable levels of internal consistency (i.e., Cronbach alphas > 0.80; [13]) for items in risk factor scales. The validity of U.S.-based CTC-YS measures and their cut points has been studied extensively and has shown good measurement properties across different age, sex, and racial and ethnic groups as well as good concurrent and predictive validity [27,28].

### 2.3. Participants

Participants in the U.S.-based sample were taken from a database of over 1,300,000 adolescents (Grades 6 to 12) from 11 different states in the U.S. The present study included only a sub-sample from students who were in grades 6, 8, and 10 as these three cohorts represented different developmental periods for prevention and validated cut points have not yet been developed for odd numbered grades (i.e., 7th, 9th, and 11th grades). Depending on the risk factor measure in question, sample sizes used in this study ranged from 138,035 to 373,985 6th-grade adolescents (*Md* = 319,477); 205,139 to 498,658 8th-grade adolescents (*Md* = 437,966); and 216,120 to 490,248 10th-grade adolescents (*Md* = 437,184). U.S.-based data were collected from 2010 to 2012 by state and local agencies. Because only aggregated data were available for this study, no information on students’ gender or age was available; however, no demographic restrictions were imposed on student participation in the surveys.

Participants in the Colombian sample were obtained from a database of over 80,000 adolescents (Grades 6 to 11) from 14 departments and 33 municipalities in Colombia. Following the U.S sub-samples, the present study included only Colombian students at grades 6th, 8th, and 10th to facilitate comparisons. Depending on the risk factor measure in question, sample sizes used in this study ranged from 3811 to 13,280 6th-grade adolescents (*Md* = 10,644); 1278 to 12,133 8th-grade adolescents (*Md* = 9569); and 2610 to 9941 10th-grade adolescents (*Md* = 7829). Colombian-based data were collected in 2012 to 2013 and 2016 to 2018 by the Corporación Nuevos Rumbos. Just over half (52.1%) of the sample was female and the average age of students was *M* = 14.0 years (*SD* = 1.76).

### 2.4. Statistical Analyses

Analyses consisted of calculating three sets of proportions of youth at “high” risk for each of the 18 risk factors: (1) the proportion of Colombian youth at “high” risk using cut points based on the distributions of risk factors observed in the Colombian data (COL_1_), (2) the proportion of Colombian youth at “high” risk using cut points observed in the U.S. data (COL_2_), and (3) the proportion of U.S. youth at “high” risk using the U.S.-based cut points (USA_2_). Each of these sets of proportions was compared with each other, by student grade (see Table 1, Table 2 and Table 3). For each comparison, odds ratios (ORs) and their 95% confidence intervals [29] were used to assess the magnitude of the difference in the pairs of proportions [30]. ORs whose 95% confidence interval did not include 1.00 were considered to be significantly (*p* < 0.05) different (i.e., an OR of 1.00 indicated that there was no difference in the proportions of youth at “high” risk for the pair in question). ORs were then transformed to Cox *d* effect sizes [31] to facilitate the interpretation of magnitudes in the differences between proportions.

**Figure 2:**
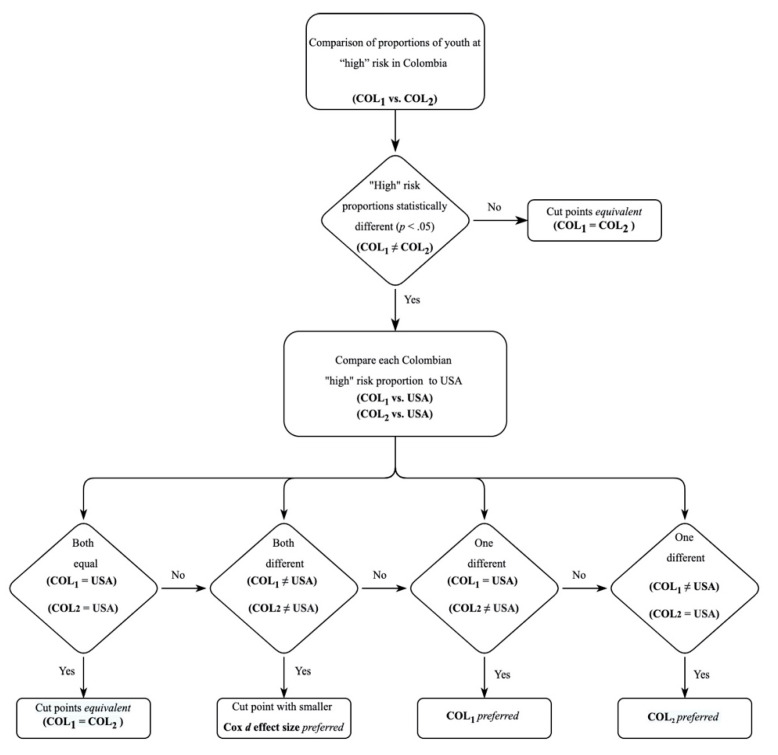
Steps for comparing the proportions of youth at “high” risk.

The procedure for comparing the proportions of youth at “high” risk followed two steps (see Figure 2). First, if there was not a statistically significant difference in the proportions of youth between COL_1_ and COL_2_, the pair of cut points were considered to have an equivalent ability to discriminate between the proportions, with indeterminant preference for Colombian-based versus U.S.-based cut points for the Colombian sample of youth. Second, if there was a statistical difference in these proportions of youth between COL_1_ versus COL_2_, then ORs obtained in comparing (a) COL_1_ versus USA_2_ and (b) COL_2_ versus USA_2_ were inspected and the comparison that showed a nonsignificant difference in their respective proportions was preferred in favor of the alternate comparison that showed a significant difference. If (a) COL_1_ versus USA_2_ and (b) COL_2_ versus USA_2_ both exhibited nonsignificant ORs, then cut points were again determined to be equivalent with indeterminant preference between the Colombian-based versus U.S.-based cut points. If (a) COL_1_ versus USA_2_ and (b) COL_2_ versus USA_2_ were both significant, the smaller difference between the proportions (as indicated by the Cox *d* effect size for the difference in pairs) was considered preferential.

For example, as shown in Table 1, for the risk factor Laws and Norms Favorable to Drug Use, 40.0% of 6th-grade youth in the Colombian sample were identified as being at “high” risk using the Colombian-based cut points (COL_1_), 86.4% of 6th-grade of youth in the Colombian sample were identified as being at “high” risk using the U.S.-based cut points (COL_2_), and 48.0% of 6th-grade youth in the U.S. sample were identified as being at “high” risk using the U.S.-based cut points (USA_2_). Comparing COL_1_ versus COL_2_ (i.e., 0.400 and 0.864, respectively) indicated that the proportions were significantly different (i.e., 95% confidence interval = 0.103–0.107) with 6th-grade youth in the Colombian sample being almost 90% (OR = 0.105) less likely to be identified as “high” risk using the U.S.-based cut points compared to the Colombian-based cut points. In this case, both (a) COL_1_ versus USA_2_ and (b) COL_2_ versus USA_2_ were statistically significant (i.e., 95% confidence intervals = 0.718–0.726 and 6.83–6.93, respectively) with *d*_Cox_ = 0.197 and 1.17, respectively. Thus, given our decision rules, the smaller *d*_Cox_ effect size for COL_1_ versus USA_2_ suggested that the Colombian-based cut points showed preferential ability to identify “high” risk youth compared to the U.S.-based cut points for this grade and risk factor.

## 3. Results

The proportions of students at “high” risk for each of the 18 examined risk factors, their ORs, 95% confidence intervals, and *d*_Cox_ effect sizes are presented in Table 1, Table 2 and Table 3, for 6th-, 8th-, and 10th-grade students, respectively. In general, the various proportions of youth considered to be at “high” risk for each risk factor showed appreciable departure from the “optimal” proportion of 0.440 that would be expected under a normal distribution for each measure, owing to the coarseness of item response options used in these measures. Significant differences in the comparisons of proportions for COL_1_ versus COL_2_ were found for 7 out of the 18 (38.9%) risk factor comparisons for 6th graders, 11 out of 18 (61.1%) comparisons for 8th graders, and 12 out of 18 (66.6%) comparisons for 10th graders. Results indicated that across the 18 examined risk factors, the Colombian-based cut points were preferable to the U.S.-based cut points in all of the 7 significant 6th-grade comparisons, all but 1 (i.e., Parental Attitudes regarding Antisocial Behavior) of the 11 significant 8th-grade comparisons, and all of the 12 significant 10th-grade comparisons.

Among the 7 significant differences in the 6th-grade comparisons of proportions of youth at “high” risk, 6 indicated that using the U.S.-based cut points would overestimate the predicted percentages of youth at “high” risk by an average difference across all risk factors of 27.3% (corresponding to an average *d*_Cox_ = 0.746). One risk factor, Low Commitment to School, showed an underestimation of 17.0% (*d*_Cox_ =0.451) by U.S.-based cut points in the observed proportion of Colombian youth at “high” risk compared to Colombian-based cut points.

Among the 11 8th-grade comparisons that demonstrated a significant difference in proportions of youth at “high” risk, 10 risk factors indicated a preference for the Colombian-based cut points, with 8 of them indicating that the U.S.-based cut points would overestimate the predicted percentages of Colombian youth at “high” risk (average difference = 17.5%; average *d*_Cox_ = 0.446), and 2 of them (Low Commitment to School and Family History of Antisocial Behavior) indicating that the U.S.-based cut points would underestimate the predicted percentages of Colombian youth at “high” risk (average difference = 22.1%; average *d*_Cox_ = 0.610). One risk factor, Parental Attitudes Favorable Towards Antisocial Behavior, indicated a preference for the use of the U.S.-based cut points, with an underestimation in the percentage of Colombian youth at “high” risk of 17.8% (*d*_Cox_ = 0.438) by the Colombian-based cut points.

Among the 12 significant 10th-grade comparisons, all favored the Colombian-based cut points, with 6 risk factors demonstrating a overestimation in the percentages of Colombian youth at “high” risk (average difference = 18.3%; average *d*_Cox_ = 0.453) and 6 risk factors demonstrating an underestimation in the percentages of Colombian youth at “high” risk (average difference = 17.8%; average *d*_Cox_ = 0.525) when using the U.S.-based cut points.

## 4. Discussion

The widespread use of epidemiologic risk and protective factor data for adolescent substance use and the selection of evidence-based prevention programs. and strategies that are based on these data, represent a significant advance in the ability to effectively discriminate “high” risk subgroups and combat this health problem. However, as is unfortunately too common in public health, the valid measurement of these constructs lags behind their operationalization. Apparently, such is the case with the international use of cut points in the distributions of risk factors for adolescent substance use, at least for the use of cut points for Colombian youth calculated from distributions of risk factors from U.S. adolescents. Results of this study point to the need for collecting large-scale national data on youth risk factors for adolescent substance use and developing country-specific cut points in the distributions of these measures. While the use of cut points in the distributions of risk factors to identify “high” risk as targets of preventive intervention is a valuable tool in local prevention programming, the placement of these cut points has important implications for research.

Results indicated that over half (55.5%) of all comparisons between the proportions of Colombian youth at “high” risk using Colombian-based cut points versus the proportion of Colombian youth at “high” risk using U.S.-based cut points showed a significant difference, and that the numbers of differences in these proportions increased as grade level increased (38.9% for 6th graders, 61.1% for 8th graders, and 66.6% for 10th graders). The primary implication of the findings from this study suggests that international comparison of youth at “high” risk may be invalidated by the use of parameters (e.g., the median of the distribution and the mean absolute deviation from median) from distributions that are not based on the population in question. Findings from this study showed a general trend for U.S.-based cut points to overestimate the proportions of Colombian youth at “high” risk in 6th and 8th grades. This was due to right-ward shifts in the distributions of most of the risk factors for these Colombian adolescents compared to the 6th- and 8th-grade samples of U.S. adolescents used in this study, which represents higher average levels of risk for these youth in Colombia than in the U.S. Consequently, a 6th- or 8th-grade youth considered to be “high” risk by U.S. standards would probably be considered to be “normal” risk by Colombian standards. For the 10th-grade samples of adolescents in this study the picture was less clear, with half of the comparisons demonstrating an overestimation in the percentages of Colombian youth at “high” risk and half of the comparisons demonstrating an underestimation in the percentages of Colombian youth at “high” risk. Interestingly, risk factors that showed an overestimation in the proportions of Colombian youth at “high” risk using U.S.-based cut points tended to be related specifically to drug use (e.g., Laws and Norms Favorable to Drug Use, Parental Attitudes for Drug Use, and Friends Use of Drugs), and risk factors that showed an underestimation in the proportions of Colombian youth at “high” risk using U.S.-based cut points tended to be non-drug specific (e.g., Family Conflict, Poor Family Management, and Rewards for Antisocial Involvement).

It is important to note that decisions made by local community-specific health coalitions and agencies in Colombia using risk profiles that were U.S.-based cut points are not necessarily invalidated by the findings from this study. The relative comparison of levels of “high” risk across an array of risk factor measures within a locality can still be useful even when the cut points that define “high” risk are consistently overestimated or underestimated. In other words, the use of “non-native” cut points may not change the selection of salient risk factors in a community as long as all the risk factors in the local profile use the same formula for determining “high” risk. The analog is as if one uses the wrong time zone in setting up a series of meetings but uses a common clock to measure the durations of the various meetings. Thus, the internationalization of the Communities That Care prevention system remains a viable approach to the effective mobilization of communities for the implementation of evidence-based prevention; however, care should be exercised to ensure that measures have the requisite cross-national equivalency.

The present study has several limitations. As the focus of this study was to compare currently existing cut points used in the U.S. and Colombia, and not to derive new cut points for this comparison, which would require individual-level data. Moreover, our secondary data sources did not provide an objective measurement for testing if cut points validly identified youth at risk (e.g., assessing sensitivity and specificity of the cut points). Another limitation of this study was not having individual-level student data in the U.S. sample (e.g., year of data collection, gender, age, and race and ethnicity) that would have allowed for a deeper psychometric comparison of U.S. and Colombian data. The derivation of county-specific cut points should be based on large samples that are as representative to the national population as possible, which also are costly and exceedingly rare. Moreover, having longitudinal data at the individual level would allow for a rigorous test of predictive validity of risk factor cut points. The large-scale samples used in this study allowed for estimation of population distributions in the U.S. and Colombia that were as representative and generalizable as possible, and consequently served to strengthen the conclusions produced in this study. Another limitation exists in the focus of this study on the relative proportions of youth identified as being at high risk given application of the Arthur et al. [11] formula for determining optimal cut points in the distributions of the CTC-YS risk factors. Our study allowed the median (*Md*) and mean absolute deviation from the median (MAD) to vary as a function of the Colombian-based data used in this study; however, we did not examine whether another formula (i.e., values different from *Md* + 0.15 × MAD) would have resulted in better sensitivity and specificity vis-a-vis a “gold standard” criterion for identifying youth at “high” risk. Such an inquiry was beyond the scope of the present study. Additionally, the study was limited by the use of youth self-report data collected via school-based administration, which may be subject to reporting and selection biases, as students may be influenced by social desirability and school populations may be impacted by high rates of school dropout. These threats to the validity of our study are tempered by the rigid data collection protocols used in both U.S. and Colombian schools with monitoring of survey completion rates and standard human subject protections, and procedures that underscore the confidentiality and anonymity of collected data; however, as school dropout in upper grades is a widespread problem in Colombia, more research is needed to ensure that school-based data are representative of their respective communities. Finally, deeper psychometric analyses that elucidate the differences found in this study are needed to fully understand the reasons behind our observed differences, for instance the under- and over-estimation of proportions of youth at “high risk” by type of risk factor (e.g., drug specific vs. not-drug specific).

## 5. Conclusions

This study is the first to statistically compare empirically derived cut points in the distributions of risk factors between large-scale samples of adolescents from multiple grades using comparable measures. Findings from this study demonstrate the need for collecting risk factor data from large-scale samples of youth in countries that rely on community assessment of prevention needs and developing country-specific cut points in the distributions of these risk factors. Although U.S.-based cut points provide a good starting point for the selection of salient risk factors for prevention programming, differences in population distributions of risk factor measures between countries can obscure the estimation of proportions of youth at “high” risk in countries that use cut points derived from non-native sources of data.

## Figures and Tables

**Table 1:** Comparison of proportions of sixth-grade students at “high” risk, odds ratios, 95% confidence intervals, and effect sizes using Colombian and United States cut-points.

Risk Factor	Proportion at “High” Risk			COL_1_ vs. COL_2_			COL_1_ vs. USA_2_			COL_2_ vs. USA_2_		
	COL_1_	COL_2_	USA_2_	OR	95% CI	*d_Cox_*	OR	95% CI	*d_Cox_*	OR	95% CI	*d_Cox_*
Laws and Norms Favorable to Drug Use	0.400	0.864	0.480	0.105	[0.103, 0.107]	1.37	0.722	[0.718, 0.726]	0.197	6.88	[6.83, 6.93]	1.17
Perceived Availability of Drugs	0.416	0.640	0.453	0.401	[0.391, 0.411]	0.554	0.860	[0.855, 0.865]	0.091	2.15	[2.13, 2.16]	0.463
Perceived Availability of Handguns	0.214	0.214	0.263	1.00	[0.933, 0.1.07]	0.000	0.763	[0.755, 0.771]	0.164	0.763	[0.755, 0.771]	0.164
Community Disorganization	0.476	0.476	0.499	1.00	[0.952, 1.05]	0.000	0.912	[0.904, 0.920]	0.056	0.912	[0.904, 0.920]	0.056
Low Commitment to School	0.450	0.280	0.211	2.10	[0.203, 2.17]	0.451	3.06	[3.03, 3.08]	0.678	1.45	[1.44, 1.47]	0.227
Parental Attitudes for Drug Use	0.311	0.311	0.114	1.00	[0.969, 1.03]	0.000	3.51	[3.47, 3.55]	0.761	3.51	[3.47, 3.55]	0.761
Parental Attitudes for ASB	0.384	0.384	0.377	1.00	[0.969, 1.03]	0.000	1.03	[1.02, 1.04]	0.018	1.03	[1.02, 1.04]	0.018
Poor Family Management	0.498	0.658	0.483	0.516	[0.503, 0.528]	0.401	1.06	[1.06, 1.07]	0.036	2.06	[2.05, 2.07]	0.438
Family History of ASB	0.314	0.854	0.480	0.078	[0.075, 0.081]	1.54	0.496	[.492, 0.500]	0.425	6.34	[6.27, 6.40]	1.12
Family Conflict	0.325	0.325	0.389	1.00	[0.957, 1.05]	0.000	0.756	[0.751, 0.726]	0.169	0.756	[0.751, 0.762]	0.169
Rewards for Antisocial Involvement	0.253	0.253	0.245	1.00	[0.938, 0.107]	0.000	1.04	[1.03, 1.05]	0.026	1.04	[1.03, 1.05]	0.026
Positive Attitudes for ASB	0.395	0.400	0.400	0.979	[0.950, 1.01]	0.013	0.979	[0.974, 0.985]	0.013	1.00	[0.994, 1.01]	0.000
Positive Attitudes for Drug Use	0.386	0.516	0.189	0.590	[0.575, 0.604]	0.320	2.70	[2.68, 2.72]	0.601	4.57	[4.54, 4.61]	0.922
Perceived Risk of Drug Use	0.430	0.550	0.445	0.617	[0.601, 0.634]	0.292	0.941	[.936, 0.946]	0.037	1.52	[1.52, 1.53]	0.255
Friends’ Use of Drugs	0.469	0.469	0.197	1.00	[0.973, 1.03]	0.000	3.60	[3.57, 3.63]	0.776	3.60	[3.57, 3.63]	0.776
Gang Involvement	0.215	0.215	0.128	1.00	[0.935, 1.07]	0.000	1.87	[1.83, 1.90]	0.378	1.87	[1.83, 1.90]	0.378
Early Initiation of Drug Use	0.194	0.194	0.234	1.00	[0.930, 1.08]	0.000	0.788	[0.780, 0.796]	0.144	.788	[0.780, 0.796]	0.144
Interaction with Antisocial Peers	0.424	0.424	0.336	1.00	[0.952, 1.05]	0.000	1.45	[1.44, 1.47]	0.227	1.45	[1.44, 1.47]	0.227

*Note.* COL_1_ = The proportion of Colombian youth at “high” risk using cut points based on the distributions of risk factors observed in the Colombian data. COL_2_ = The proportion of Colombian youth at “high” risk using cut points observed in the U.S. data. USA_2_ = The proportion of U.S. youth at “high” risk using the U.S.-based cut points. ASB = antisocial behavior. OR= odds ratio. CI = confidence interval. *d_Co_*_x_ = Cox effect size.

**Table 2:** Comparison of proportions of eight-grade students at “high” risk, odds ratios, 95% confidence intervals, and effect sizes using Colombian and United States cut points.

Risk Factor	Proportion at “High” Risk			COL_1_ vs. COL_2_			COL_1_ vs. USA_2_			COL_2_ vs. USA_2_		
	COL_1_	COL_2_	USA_2_	OR	95% CI	*d_Cox_*	OR	95% CI	*d_Cox_*	OR	95% CI	*d_Cox_*
Laws and Norms Favorable to Drug Use	0.467	0.698	0.400	0.379	[0.371, 0.388]	0.588	1.31	[1.31, 1.32]	0.072	3.47	[3.45, 3.49]	0.327
Perceived Availability of Drugs	0.464	0.564	0.454	0.669	[0.651, 0.688]	0.243	1.04	[1.04, 105]	0.011	1.56	[1.55, 1.56]	0.116
Perceived Availability of Handguns	0.357	0.357	0.367	1.00	[0.946, 0.1.06]	0.000	0.958	[0.801, 1.14]	0.011	0.958	[0.801, 1.14]	0.011
Community Disorganization	0.394	0.530	0.436	0.577	[0.550, 0.605]	0.334	0.841	[0.834, 0.848]	0.046	1.46	[1.45, 1.47]	0.099
Low Commitment to School	0.451	0.279	0.462	2.12	[0.205, 2.20]	0.456	0.957	[0.952, 961]	0.012	0.451	[0.448, 0.453]	0.210
Parental Attitudes for Drug Use	0.492	0.492	0.237	1.00	[0.974, 1.03]	0.000	3.12	[3.10, 3.14]	0.299	3.12	[3.10, 3.14]	0.299
Parental Attitudes for ASB	0.378	0.556	0.491	0.485	[0.472, 0.499]	0.438	0.630	[6.27, 6.33]	0.122	1.30	[1.29, 1.30]	0.069
Poor Family Management	0.449	0.522	0.473	0.746	[0.726, 0.767]	0.177	0.908	[0.903, 0.913]	0.025	1.22	[1.21, 1.22]	0.052
Family History of ASB	0.473	0.203	0.456	3.52	[3.26, 3.81]	0.763	1.07	[1.06, 1.08]	0.018	0.304	[0.301, 0.307]	0.314
Family Conflict	0.409	0.409	0.493	1.00	[0.960, 1.04]	0.000	0.712	[0.707, 0.716]	0.090	0.712	[0.707, 0.716]	0.090
Rewards for Antisocial Involvement	0.461	0.461	0.456	1.00	[0.952, 0.105]	0.000	1.02	[1.01, 1.03]	0.005	1.02	[1.01, 1.03]	0.005
Positive Attitudes for ASB	0.441	0.441	0.468	1.00	[0.970, 1.03]	0.000	0.897	[0.892, 0.901]	0.029	0.897	[0.994, 1.01]	0.029
Positive Attitudes for Drug Use	0.484	0.775	0.437	0.272	[0.267, 0.278]	0.788	1.21	[1.20, 1.21]	0.050	4.44	[4.41, 4.46]	0.392
Perceived Risk of Drug Use	0.382	0.511	0.379	0.592	[0.574, 0.609]	0.318	1.01	[1.01, 1.02]	0.003	1.71	[1.70, 1.72]	0.142
Friends’ Use of Drugs	0.453	0.728	0.479	0.309	[0.302, 0.317]	0.711	0.901	[0.896, 0.905]	0.028	2.91	[2.90, 2.93]	0.281
Gang Involvement	0.226	0.226	0.181	1.00	[0.934, 1.07]	0.000	1.32	[1.30, 1.34]	0.073	1.32	[1.30, 1.34]	0.073
Early Initiation of Drug Use	0.320	0.320	0.451	1.00	[0.939, 1.06]	0.000	0.573	[0.566, 0.579]	0.147	0.573	[0.566, 0.579]	0.147
Interaction with Antisocial Peers	0.391	0.559	0.448	0.507	[0.484, 0.530]	0.412	0.791	[0.786, 0.796]	0.062	1.56	[1.55, 1.57]	0.117

*Note.* COL_1_ = The proportion of Colombian youth at “high” risk using cut points based on the distributions of risk factors observed in the Colombian data. COL_2_ = The proportion of Colombian youth at “high” risk using cut points observed in the U.S. data. USA_2_ = The proportion of U.S. youth at “high” risk using the U.S.-based cut points. ASB = antisocial behavior. OR= odds ratio. CI = confidence interval. *d_Co_*_x_ = Cox effect size.

**Table 3:** Comparison of proportions of tenth-grade students at “high” risk, odds ratios, 95% confidence intervals, and effect sizes using Colombian and United States cut points.

Risk Factor	Proportion at “High” Risk			COL_1_ vs. COL_2_			COL_1_ vs. USA_2_			COL_2_ vs. USA_2_		
	COL_1_	COL_2_	USA_2_	OR	95% CI	*d_Cox_*	OR	95% CI	*d_Cox_*	OR	95% CI	*d_Cox_*
Laws and Norms Favorable to Drug Use	0.454	0.694	0.484	0.367	[0.357, 0.376]	0.608	0.886	[0.882, 0.891]	0.073	2.42	[2.40, 2.43]	0.535
Perceived Availability of Drugs	0.441	0.441	0.475	1.00	[0.966, 1.04]	0.000	0.872	[0.867, 0.877]	0.083	0.872	[0.867, 0.877]	0.083
Perceived Availability of Handguns	0.433	0.433	0.455	1.00	[0.946, 1.06]	0.000	0.915	[0.908, 0.921]	0.054	0.915	[0.908, 0.921]	0.054
Community Disorganization	0.467	0.615	0.468	0.548	[0.522, 0.576]	0.364	0.996	[0.987, 1.00]	0.002	1.82	[1.80, 1.83]	0.362
Low Commitment to School	0.407	0.227	0.487	2.34	[2.24, 2.44]	0.515	0.723	[0.719, 0.727]	0.197	0.309	[0.308, 0.311]	0.711
Parental Attitudes for Drug Use	0.420	0.634	0.396	0.418	[0.407, 0.429]	0.529	1.10	[1.10, 1.11]	0.060	2.64	[2.63, 2.66]	0.589
Parental Attitudes for ASB	0.377	0.377	0.349	1.00	[0.964, 1.04]	0.000	1.13	[1.12, 1.14]	0.073	1.13	[1.12, 1.14]	0.073
Poor Family Management	0.470	0.389	0.493	1.39	[1.34, 1.44]	0.201	0.912	[0.907, 0.917]	0.056	0.655	[0.651, 0.658]	0.257
Family History of ASB	0.480	0.092	0.478	9.11	[8.00, 10.4]	1.339	1.01	[1.00, 1.02]	0.005	0.111	[0.109, 0.112]	1.33
Family Conflict	0.446	0.274	0.399	2.13	[2.02, 2.25]	0.459	1.21	[1.20, 1.22]	0.117	0.568	[0.565, 0.572]	0.342
Rewards for Antisocial Involvement	0.448	0.333	0.421	1.63	[1.53, 1.73]	0.294	1.12	[1.11, 1.12]	0.067	0.687	[0.682, 0.692]	0.228
Positive Attitudes for ASB	0.446	0.314	0.410	1.76	[1.70, 1.83]	0.342	1.16	[1.15, 1.16]	0.089	0.659	[0.655, 0.662]	0.253
Positive Attitudes for Drug Use	0.448	0.628	0.453	0.481	[4.68, 4.93]	0.444	0.980	[0.975, 0.985]	0.012	2.04	[4.54, 4.61]	0.432
Perceived Risk of Drug Use	0.441	0.441	0.475	1.00	[0.966, 1.04]	0.000	0.872	[0.867, 0.877]	0.083	0.872	[0.867, 0.877]	0.083
Friends’ Use of Drugs	0.465	0.602	0.481	0.575	[0.559, 0.559]	0.336	0.938	[0.933, 0.943]	0.039	1.63	[1.62, 1.64]	0.297
Gang Involvement	0.195	0.195	0.183	1.00	[0.922, 1.08]	0.000	1.08	[1.07, 1.10]	0.047	1.08	[1.07, 1.10]	0.047
Early Initiation of Drug Use	0.362	0.362	0.632	1.00	[0.941, 1.06]	0.000	0.330	[0.332, 0.328]	0.671	0.330	[0.332, 0.328]	0.671
Interaction with Antisocial Peers	0.370	0.546	0.455	0.488	[0.464, 0.513]	0.434	0.703	[0.699, 0.708]	0.213	1.44	[1.43, 1.45]	0.221

*Note.* COL_1_ = The proportion of Colombian youth at “high” risk using cut points based on the distributions of risk factors observed in the Colombian data. COL_2_ = The proportion of Colombian youth at “high” risk using cut points observed in the U.S. data. USA_2_ = The proportion of U.S. youth at “high” risk using the U.S.-based cut points. ASB = antisocial behavior. OR= odds ratio. CI = confidence interval. *d_Co_*_x_ = Cox effect size.

## Data Availability

The aggregated data that support the findings of this study are available from the corresponding author (E.C.B), upon reasonable request to the firm of Bach Harrison L.L.C., Survey Research and Evaluation Services (http://www.bach-harrison.com/), the Corporación Nuevos Rumbos (https://www.nuevosrumbos.org/), and the Universidad El Bosque, in Bogotá, Colombia (https://www.unbosque.edu.co/).

## Supplementary Materials

The following are available online at https://www.mdpi.com/1660-4601/18/2/470/s1, Table S1: Risk Factors Items and Cut Points Construct Dictionary.
