# Supplementary material for: A Comparison of Cut Points for Measuring Risk Factors for Adolescent Substance Use and Antisocial Behaviors in the U.S. and Colombia
Source: Int J Environ Res Public Health. 2021 Jan 6;18(2):470. doi: 10.3390/ijerph18020470 (PMC7827061; doi:10.3390/ijerph18020470)
Supplement: Supplementary file 1 [file ijerph-18-00470-s001.pdf]

Risk factors Items and Cut Points

Construct Dictionary

Annex 1

COMMUNITY RISK FACTOR: Community Disorganization

CRCDO (4 point scale) | *alpha=0.828*

CUT POINTS: 6th grade (COL= 1.676; USA = 1.690) | 8th grade (COL = 2.057; USA = 1.884) | 10th grade (COL = 2.601; USA = 2.628)

Variable Name	Question	Scoring
SAFENH	I feel safe in my neighborhood.	NO! (4) no (3) yes (2) YES! (1)
	How much do each of the following statements describe your neighborhood?	
NHCRIME	Crime and/or drug selling.	NO! (1) no (2) yes (3) YES! (4)
NHFIGHT	Fights.	NO! (1) no (2) yes (3) YES! (4)
NHEMPTY	Lots of empty or abandoned buildings.	NO! (1) no (2) yes (3) YES! (4)
NHGRAFF	Lots of graffiti.	NO! (1) no (2) yes (3) YES! (4)

COMMUNITY RISK FACTOR: Perceived Availability of Drugs

CRPAD (4 point scale) | *alpha=0.867*

CUT POINTS: 6th grade (COL = 1.588); USA = 1.064) | 8th grade (COL = 2.104; USA = 1.861) | 10th grade (COL = 1.879; USA = 2.081)

Variable Name	Question	Scoring
GETCIG	If you wanted to get some cigarettes, how easy would it be for you to get some?	Very Hard (1) Sort of Hard (2) Sort of Easy (3) Very Easy (4)
GETALC	If you wanted to get some beer, wine or hard liquor (for example, vodka, whiskey or gin), how easy would it be for you to get some?	Very Hard (1) Sort of Hard (2) Sort of Easy (3) Very Easy (4)
GETMAR	If you wanted to get some marijuana, how easy would it be for you to get some?	Very Hard (1) Sort of Hard (2) Sort of Easy (3) Very Easy (4)
GETDRUG	If you wanted to get a drug like cocaine, LSD, or amphetamines, how easy would it be for you to get some?	Very Hard (1) Sort of Hard (2) Sort of Easy (3) Very Easy (4)

Note. COL = Cut point based on the distributions of risk factors observed in the Colombian data; USA = U.S.-based cut point

COMMUNITY RISK FACTOR: Perceived Availability of Handguns

CRPAG (4 point scale) | *alpha=N/A*

CUT POINTS: 6th grade (COL = 1.053; USA = 1.068) | 8th grade (COL = 1.084; USA = 1.097) | 10th grade (COL = 1.101; USA = 1.136)

Variable Name	Question	Scoring
GETGUN	If you wanted to get a handgun, how easy would it be for you to get one?	Very Hard (1) Sort of Hard (2) Sort of Easy (3) Very Easy (4)

COMMUNITY RISK FACTOR: Laws and Norms Favorable to Drug Use

CRLNFD (4 point scale) | *alpha=0.823*

CUT POINTS: 6th grade (COL = 2.2106; USA = 1.568) | 8th grade (COL = 2.344; USA = 2.076) | 10th grade (COL = 2.481; USA = 2.277)

Variable Name	Question	Scoring
POLALC	If a kid drank some beer, wine or hard liquor (for example, vodka, whiskey or gin) in your neighborhood would he or she be caught by the police?	NO! (4) no (3) yes (2) YES! (1)
POLMAR	If a kid smoked marijuana in your neighborhood would he or she be caught by the police?	NO! (4) no (3) yes (2) YES! (1)
POLGUN	If a kid carried a handgun in your neighborhood would he or she be caught by the police?	NO! (4) no (3) yes (2) YES! (1)
POLCIG	If a kid smoked a cigarette in your neighborhood would he or she be caught by the police?*	NO! (4) no (3) yes (2) YES! (1)
	How wrong would most adults (over 21) in your neighborhood think it is for kids your age…	
AWRMAR	…to use marijuana?	Very Wrong (1) Wrong (2) A Little Bit Wrong (3) Not Wrong at All (4)
AWRALC	…to drink alcohol?	Very Wrong (1) Wrong (2) A Little Bit Wrong (3) Not Wrong at All (4)
AWRCIG	…to smoke cigarettes?	Very Wrong (1) Wrong (2) A Little Bit Wrong (3) Not Wrong at All (4)

Note. COL = Cut point based on the distributions of risk factors observed in the Colombian data; USA = U.S.-based cut point

FAMILY RISK FACTOR: Family History of Antisocial Behavior

FRFAB (5 point scale) | *alpha=0.825*

CUT POINTS: 6th grade (COL = 1.468; USA = 1.362) | 8th grade (COL = 1.477; USA = 1.710) | 10th grade (COL = 1.533; USA = 2.131)

Variable Name	Question	Scoring				
FAMPROB	Has anyone in your family ever had a severe alcohol or drug problem?	No (1)	Yes (5)			
	Have any of your brothers or sisters ever…					
SIBALC	…drunk beer, wine or hard liquor (for example, vodka, whiskey or gin)?	No (1)	Yes (5)	I Don't Have Any Brothers or Sisters (0)		
SIBMAR	…smoked marijuana?	No (1)	Yes (5)	I Don't Have Any Brothers or Sisters (0)		
SIBCIG	…smoked cigarettes?	No (1)	Yes (5)	I Don't Have Any Brothers or Sisters (0)		
SIBGUN	…taken a handgun to school?	No (1)	Yes (5)	I Don't Have Any Brothers or Sisters (0)		
SIBSUS	…been suspended or expelled from school?	No (1)	Yes (5)	I Don't Have Any Brothers or Sisters (0)		
	About how many adults (over 21) have you known personally who in the past year have…					
ADUDRUG	…used marijuana, crack, cocaine, or other drugs?	None (1)	1 adult (2)	2 adults (3)	3 or 4 adults (4)	5 or more adults (5)
ADSDRUG	…sold or dealt drugs?	None (1)	1 adult (2)	2 adults (3)	3 or 4 adults (4)	5 or more adults (5)
ADSTEAL	…done other things that could get them in trouble with the police like stealing, selling stolen goods, mugging or assaulting others, etc.	None (1)	1 adult (2)	2 adults (3)	3 or 4 adults (4)	5 or more adults (5)
ADDRUNK	…gotten drunk or high?	None (1)	1 adult (2)	2 adults (3)	3 or 4 adults (4)	5 or more adults (5)

Note. COL = Cut point based on the distributions of risk factors observed in the Colombian data; USA = U.S.-based cut point

FAMILY RISK FACTOR: Poor Family Management

FRPFM (4 point scale) | *alpha=0.857*

CUT POINTS: 6th grade (COL = 1.568; USA = 1.312) | 8th grade (COL = 1.814; USA_2=1.701) | 10th grade (COL = 1.811; USA = 1.953)

Variable Name	Question	Scoring
FAMRULE	The rules in my family are clear.	NO! (4) no (3) yes (2) YES! (1)
HMWORK	My parents ask if I've gotten my homework done.	NO! (4) no (3) yes (2) YES! (1)
PARKNOW	When I am not at home, one of my parents knows where I am and who I am with.	NO! (4) no (3) yes (2) YES! (1)
CMHOME	Would your parents know if you did not come home on time?	NO! (4) no (3) yes (2) YES! (1)
CLRRULE	My family has clear rules about alcohol and drug use.	NO! (4) no (3) yes (2) YES! (1)
CATCHAL	If you drank some beer or wine or hard liquor (for example, vodka, whiskey or gin) without your parents' permission, would you be caught by your parents?	NO! (4) no (3) yes (2) YES! (1)
CATCHGN	If you carried a handgun without your parents' permission, would you be caught by your parents?	NO! (4) no (3) yes (2) YES! (1)
CATCHSK	If you skipped school, would you be caught by your parents?	NO! (4) no (3) yes (2) YES! (1)

FAMILY RISK FACTOR: Family Conflict

FRFC (4 point scale) | *alpha=0.804*

CUT POINTS: 6th grade (COL = 2.084; USA = 2.099) | 8th grade (COL =2 .084; USA = 2.098) | 10th grade (COL = 2.074; USA = 2.427)

Variable Name	Question	Scoring
ARGUE	We argue about the same things in my family over and over.	NO! (1) no (2) yes (3) YES! (4)
SERARG	People in my family have serious arguments.	NO! (1) no (2) yes (3) YES! (4)
FAMYELL	People in my family often insult or yell at each other.	NO! (1) no (2) yes (3) YES! (4)

Note. COL = Cut point based on the distributions of risk factors observed in the Colombian data; USA = U.S.-based cut point

FAMILY RISK FACTOR: Parental Attitudes Favorable Toward Drug Use

FRPFD (4 point scale) | *alpha=0.799*

CUT POINTS: 6th grade (COL = 1.036; USA = 1 .013) | 8th grade (COL = 1.053; USA = 1.029) | 10th grade (COL = 1.388; USA = 1.064)

Variable Name	Question	Scoring			
	How wrong do your parents feel it would be for you to…				
WRPRALC	…drink beer, wine or hard liquor (for example, vodka, whiskey or gin) regularly (at least once or twice a month)?	Very Wrong (1)	Wrong (2)	A Little Bit Wrong (3)	Not Wrong at All (4)
WRPRCIG	…smoke cigarettes?	Very Wrong (1)	Wrong (2)	A Little Bit Wrong (3)	Not Wrong at All (4)
WRPRMAR	…smoke marijuana?	Very Wrong (1)	Wrong (2)	A Little Bit Wrong (3)	Not Wrong at All (4)

FAMILY RISK FACTOR: Parental Attitudes Favorable to Antisocial Behavior

FRPAB (4 point scale) | *alpha=0.733*

CUT POINTS: 6th grade (COL = 1.048; USA = 1.040) | 8th grade (COL = 1.397; USA_2 = 1.059) | 10th grade (COL = 1.393; USA = 1.396)

Variable Name	Question	Scoring			
	How wrong do your parents feel it would be for you to…				
WRPRSTL	…steal something worth more than $5?	Very Wrong (1)	Wrong (2)	A Little Bit Wrong (3)	Not Wrong at All (4)
WRPRDRW	…draw graffiti, or write things or draw pictures on buildings or other property (without the owner's permission)?	Very Wrong (1)	Wrong (2)	A Little Bit Wrong (3)	Not Wrong at All (4)
WRPRFGT	…pick a fight with someone?	Very Wrong (1)	Wrong (2)	A Little Bit Wrong (3)	Not Wrong at All (4)

Note. COL = Cut point based on the distributions of risk factors observed in the Colombian data; USA = U.S.-based cut point

SCHOOL RISK FACTOR: Low Commitment to School

SRLCS (4 point scale) | *alpha=0.793*

CUT POINTS: 6th grade (COL = 1.781; USA = 2.075) | 8th grade (COL = 2.071; USA = 2.365) | 10th grade (COL = 2.065; USA = 2.509)

Variable Name	Question	Scoring
MISSC	During the LAST FOUR WEEKS how many whole days of school have you missed because you skipped or "cut"?	None (1) 1 (1.67) 2 (2.33) 3 (3) 4-5 (3.67) 6-10 (4.33) 11 or more (5)
SCHIMP	How often do you feel that the schoolwork you are assigned is meaningful and important?	Never (5) Seldom (4) Sometimes (3) Often (2) Almost Always (1)
SCHINT	How interesting are most of your courses to you?	Very Interesting And stimulating (1) Quite Interesting (2) Fairly Interesting (3) Slightly Boring (4) Very Boring (5)
SCHLRN	How important do you think the things you are learning in school are going to be for your later life?	Very Important (1) Quite Important (2) Fairly Important (3) Slightly Important (4) Not at all Important (5)
	Now, thinking back over the past year in school, how often did you…	
ENJSCH	…enjoy being in school?	Never (5) Seldom (4) Sometimes (3) Often (2) Almost Always (1)
HTSCH	…hate being in school?	Never (1) Seldom (2) Sometimes (3) Often (4) Almost Always (5)
BWSCH	…try to do your best work in school?	Never (5) Seldom (4) Sometimes (3) Often (2) Almost Always (1)

Note. COL = Cut point based on the distributions of risk factors observed in the Colombian data; USA = U.S.-based cut point

PEER-INDIVIDUAL RISK FACTOR: Gang Involvement

PRGI (9 point scale) |*alpha=0.873*

CUT POINTS: 6th grade (COL = 0.06; USA = 0.041) | 8th grade (COL = 0.071; USA = 0.063) | 10th grade (COL = 0.063; USA = 0.057)

Variable Name	Question	Scoring
GANG	Have you ever belonged to a gang?	No (0) Yes (8)
GANGNM	If you have ever belonged to a gang, did the gang have a name?	No (1) Yes (8) I have never belonged to a gang (0)
	In the past year (12 months), how many of your best friends have…	
FRGANG	…been members of a gang?	None of my friends (0); 1 of my friends (1); 2 of my friends (2); 3 of my friends (3); 4 of my friends (4)F
	***How old were you when you* first…**	
AGEGANG	…belonged to a gang?	Never Have (0); 10 or Younger (8); 11 (7); 12 (6); 13 (5); 14 (4); 15 (3); 16 (2); 17 or Older (1)

PEER-INDIVIDUAL RISK FACTOR: Perceived Risks of Drug Use

PRPRD (4 point scale) |*alpha=0.820*

CUT POINTS: 6th grade (COL = 1.781; USA = 2.075) | 8th grade (COL =2.071; USA = 2.365) | 10th grade (COL = 2.065; USA = 2.509)

Variable Name	Question	Scoring
	How much do you think people risk harming themselves (physically or in other ways) if they…	
HMCIG	…smoke one or more packs of cigarettes per day?	No risk (4) Slight Risk (3) Moderate Risk (2) Great Risk (1)
HMMARO	…try marijuana once or twice?	No risk (4) Slight Risk (3) Moderate Risk (2) Great Risk (1)
HMMARR	…smoke marijuana regularly?	No risk (4) Slight Risk (3) Moderate Risk (2) Great Risk (1)
HMALC	…take one or two drinks of an alcoholic beverage (beer, wine, or liquor) nearly every day?	No risk (4) Slight Risk (3) Moderate Risk (2) Great Risk (1)

Note. COL = Cut point based on the distributions of risk factors observed in the Colombian data; USA = U.S.-based cut point

PEER-INDIVIDUAL RISK FACTOR: Early Initiation of Drug Use

PREID (9-point scale) | *alpha=0.801*

CUT POINTS: 6th grade (COL = 0.091; USA = 0.093) | 8th grade (COL = 0.124; USA = 0.194) | 10th grade (COL = 0.121; USA = 0.111)

Variable Name	Question	Scoring								
	How old were you when you first…									
AGECIG	…smoked a cigarette, even just a puff?	Never Have (0)	10 or Younger (8)	11 (7)	12 (6)	13 (5)	14 (4)	15 (3)	16 (2)	17 or Older (1)
AGEALC	…had more than a sip or two of beer, wine or hard liquor (for example, vodka, whiskey or gin)?	Never Have (0)	10 or Younger (8)	11 (7)	12 (6)	13 (5)	14 (4)	15 (3)	16 (2)	17 or Older (1)
AGEALCR	…began drinking alcoholic beverages regularly, that is, at least once or twice a month?	Never Have (0)	10 or Younger (8)	11 (7)	12 (6)	13 (5)	14 (4)	15 (3)	16 (2)	17 or Older (1)
AGEMAR	…smoked marijuana?	Never Have (0)	10 or Younger (8)	11 (7)	12 (6)	13 (5)	14 (4)	15 (3)	16 (2)	17 or Older (1)

PEER-INDIVIDUAL RISK FACTOR: Favorable Attitudes Toward Drug Use

PRFAD (4-point scale) | *alpha=0.864*

CUT POINTS: 6th grade (COL = 1.314; USA = 1.020) | 8th grade (COL = 1.578; USA = 1.060) | 10th grade (COL = 1.825; USA = 1.594)

Variable Name	Question	Scoring			
	How wrong do you think it is for someone your age to…				
WRALC	…drink beer, wine or hard liquor (for example, vodka, whiskey or gin) regularly, that is, at least once or twice a month?	Very Wrong (1)	Wrong (2)	A Little Bit Wrong (3)	Not Wrong at All (4)
WRCIG	…smoke cigarettes?	Very Wrong (1)	Wrong (2)	A Little Bit Wrong (3)	Not Wrong at All (4)
WRMAR	…smoke marijuana?	Very Wrong (1)	Wrong (2)	A Little Bit Wrong (3)	Not Wrong at All (4)
WRDRUG	…use LSD, cocaine, amphetamines or another illegal drug?	Very Wrong (1)	Wrong (2)	A Little Bit Wrong (3)	Not Wrong at All (4)

Note. COL = Cut point based on the distributions of risk factors observed in the Colombian data; USA = U.S.-based cut point

PEER-INDIVIDUAL RISK FACTOR: Favorable Attitudes Toward Antisocial Behavior

PRATA (4 point scale) | *alpha=0.829*

CUT POINTS: 6th grade (COL = 1.257; USA = 1.248) | 8th grade (COL = 1.465 USA = 1.467) | 10th grade (COL = 1.462; USA = 1.668)

Variable Name	Question	Scoring			
	How wrong do you think it is for someone your age to…				
WRGUN	…take a handgun to school?	Very Wrong (1)	Wrong (2)	A Little Bit Wrong (3)	Not Wrong at All (4)
WRSTL	…steal something worth more than $5?	Very Wrong (1)	Wrong (2)	A Little Bit Wrong (3)	Not Wrong at All (4)
WRATT	…attack someone with the idea of seriously hurting them?	Very Wrong (1)	Wrong (2)	A Little Bit Wrong (3)	Not Wrong at All (4)
WRFGT	…pick a fight with someone?	Very Wrong (1)	Wrong (2)	A Little Bit Wrong (3)	Not Wrong at All (4)
WRSKIP	…stay away from school all day when their parents think they are at school?	Very Wrong (1)	Wrong (2)	A Little Bit Wrong (3)	Not Wrong at All (4)

PEER-INDIVIDUAL RISK FACTOR: Friends' Use of Drugs

PRFUD (5 point scale) | *alpha=0.853*

CUT POINTS: 6th grade (COL = 0.078; USA = 0.025) | 8th grade (COL = 0.872; USA = 0.091) | 10th grade (COL = 1.123; USA = 0.895)

Variable Name	Question	Scoring				
	In the past year (12 months), how many of your best friends have…					
FRCIG	…smoked cigarettes?	None of my friends (0)	1 of my friends (1)	2 of my friends (2)	3 of my friends (3)	4 of my friends (4)
FRALC	…tried beer, wine or hard liquor (for example, vodka, whiskey or gin) when their parents didn't know about it?	None of my friends (0)	1 of my friends (1)	2 of my friends (2)	3 of my friends (3)	4 of my friends (4)
FRMAR	…used marijuana?	None of my friends (0)	1 of my friends (1)	2 of my friends (2)	3 of my friends (3)	4 of my friends (4)
FRDRUG	…used LSD, cocaine, amphetamines or other illegal drugs?	None of my friends (0)	1 of my friends (1)	2 of my friends (2)	3 of my friends (3)	4 of my friends (4)

Note. COL = Cut point based on the distributions of risk factors observed in the Colombian data; USA = U.S.-based cut point

PEER-INDIVIDUAL RISK FACTOR: Rewards for Antisocial Involvement

PRRAI (5-point scale) | *alpha=0.841*

CUT POINTS: 6th grade (COL = 1.044; USA = 1.040) | 8th grade (COL = 1.084; USA = 1.086) | 10th grade (COL = 1.347; USA = 1.782)

Variable Name	Question	Scoring				
	What are the chances you would be seen as cool if you…					
COOLCIG	…smoked cigarettes?	No or Very Little Chance (1)	Little Chance (2)	Some Chance (3)	Pretty Good Chance (4)	Very Good Chance (5)
COOLALC	…began drinking alcoholic beverages regularly, that is, at least once or twice a month?	No or Very Little Chance (1)	Little Chance (2)	Some Chance (3)	Pretty Good Chance (4)	Very Good Chance (5)
COOLMAR	…smoked marijuana?	No or Very Little Chance (1)	Little Chance (2)	Some Chance (3)	Pretty Good Chance (4)	Very Good Chance (5)
COOLGUN	…carried a handgun?	No or Very Little Chance (1)	Little Chance (2)	Some Chance (3)	Pretty Good Chance (4)	Very Good Chance (5)

PEER-INDIVIDUAL RISK FACTOR: Interaction with Antisocial Peers

PRIAP (5-point scale) | *alpha=0.784*

CUT POINTS: 6th grade (COL = 0.037; USA = 0.023) | 8th grade (COL = 0.219; USA = 0.040) | 10th grade (COL = 0.215; USA = 0.046)

Variable Name	Question	Scoring				
	In the past year (12 months), how many of your best friends have…					
FRSUS	…been suspended from school?	None of my friends (0)	1 of my friends (1)	2 of my friends (2)	3 of my friends (3)	4 of my friends (4)
FRGUN	…carried a handgun?	None of my friends (0)	1 of my friends (1)	2 of my friends (2)	3 of my friends (3)	4 of my friends (4)
FRSOLD	…sold illegal drugs?	None of my friends (0)	1 of my friends (1)	2 of my friends (2)	3 of my friends (3)	4 of my friends (4)
FRSTL	…stolen or tried to steal a motor vehicle such as a car or motorcycle?	None of my friends (0)	1 of my friends (1)	2 of my friends (2)	3 of my friends (3)	4 of my friends (4)
FRARST	…been arrested?	None of my friends (0)	1 of my friends (1)	2 of my friends (2)	3 of my friends (3)	4 of my friends (4)
FRDROP	…dropped out of school?	None of my friends (0)	1 of my friends (1)	2 of my friends (2)	3 of my friends (3)	4 of my friends (4)

Note. COL = Cut point based on the distributions of risk factors observed in the Colombian data; USA = U.S.-based cut point
